# Decomposition of Picolyl Radicals at High Temperature: A Mass Selective Threshold Photoelectron Spectroscopy Study

**DOI:** 10.1002/chem.201903937

**Published:** 2019-12-05

**Authors:** Engelbert Reusch, Fabian Holzmeier, Marius Gerlach, Ingo Fischer, Patrick Hemberger

**Affiliations:** ^1^ Institute of Physical and Theoretical Chemistry University of Würzburg Am Hubland Süd 97074 Würzburg Germany; ^2^ Dipartimento di Fisica Politecnico di Milano Piazza Leonardo da Vinci 32 20133 Milano Italy; ^3^ Laboratory for Femtochemistry and Synchrotron Radiation Paul Scherrer Institut (PSI) 5232 Villigen Switzerland

**Keywords:** ionization energy, photoelectron spectroscopy, pyrolysis, radicals, synchrotron radiation

## Abstract

The reaction products of the picolyl radicals at high temperature were characterized by mass‐selective threshold photoelectron spectroscopy in the gas phase. Aminomethylpyridines were pyrolyzed to initially produce picolyl radicals (*m*/*z*=92). At higher temperatures further thermal reaction products are generated in the pyrolysis reactor. All compounds were identified by mass‐selected threshold photoelectron spectroscopy and several hitherto unexplored reactive molecules were characterized. The mechanism for several dissociation pathways was outlined in computations. The spectrum of *m*/*z*=91, resulting from hydrogen loss of picolyl, shows four isomers, two ethynyl pyrroles with adiabatic ionization energies (IE_ad_) of 7.99 eV (2‐ethynyl‐1*H*‐pyrrole) and 8.12 eV (3‐ethynyl‐1*H*‐pyrrole), and two cyclopentadiene carbonitriles with IE′s of 9.14 eV (cyclopenta‐1,3‐diene‐1‐carbonitrile) and 9.25 eV (cyclopenta‐1,4‐diene‐1‐carbonitrile). A second consecutive hydrogen loss forms the cyanocyclopentadienyl radical with IE′s of 9.07 eV (*T*_0_) and 9.21 eV (S_1_). This compound dissociates further to acetylene and the cyanopropynyl radical (IE=9.35 eV). Furthermore, the cyclopentadienyl radical, penta‐1,3‐diyne, cyclopentadiene and propargyl were identified in the spectra. Computations indicate that dissociation of picolyl proceeds initially via a resonance‐stabilized seven‐membered ring.

## Graphical abstract

**The picolyl radical** is a crucial intermediate in reactive environments, for example, the combustion of biofuels. Its unimolecular dissociation products at high temperature were characterized by mass‐selective threshold photoelectron spectroscopy and several hitherto unexplored reactive molecules were characterized.

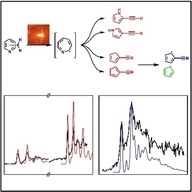

## Introduction

**Scheme 1:**
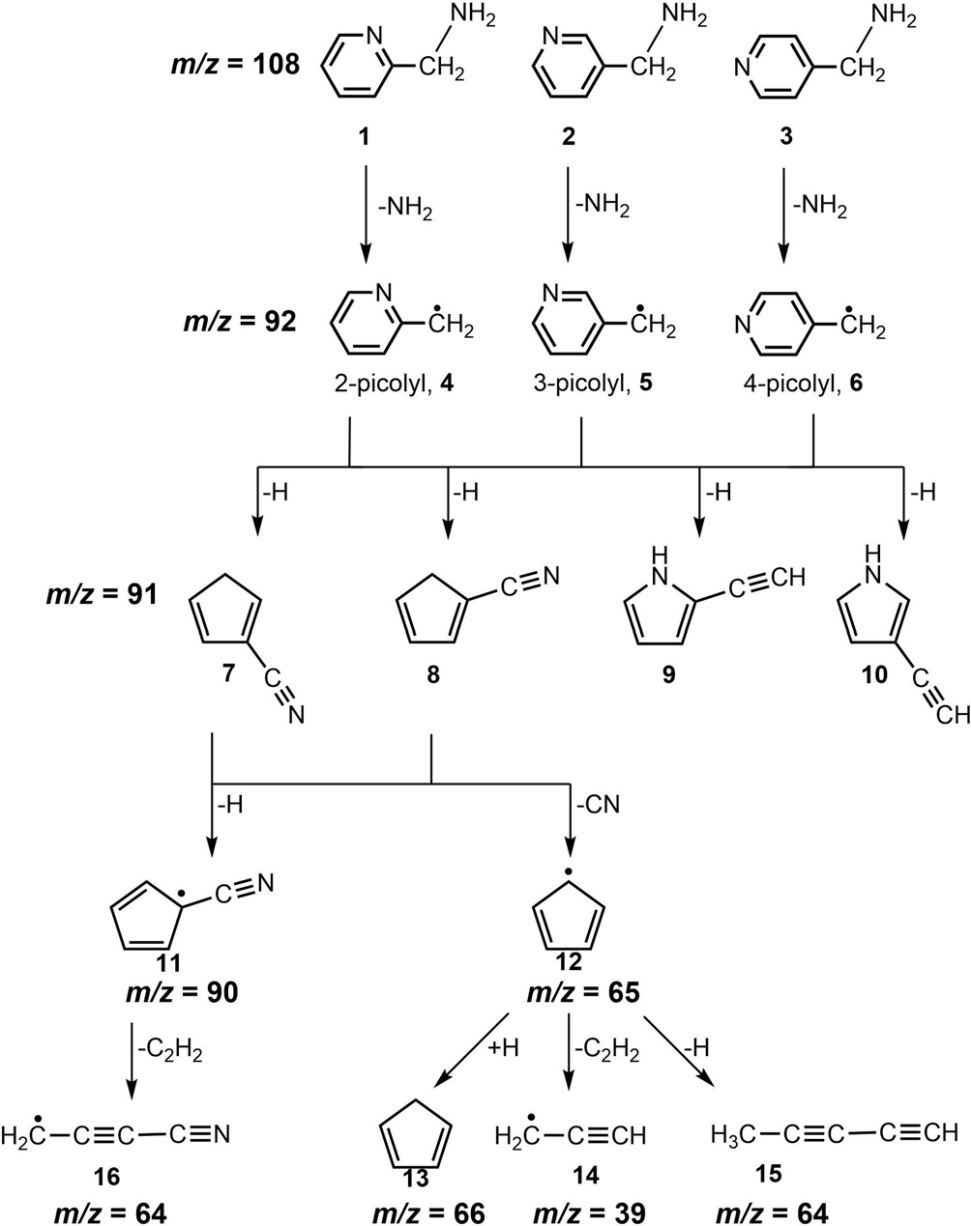
Identified products and possible pyrolysis pathway of the three aminomethylpyridines **1**, **2** and **3**.

Recently we investigated the photoionization of the three picolyl isomers, designated **4**, **5** and **6** in Scheme 1, employing mass selective threshold photoelectron spectroscopy (ms‐TPES) and obtained ionization energies (IE) of 7.70±0.02, 7.59±0.01 and 8.01±0.01 eV for **4**, **5** and **6**.^1^ In addition, a vibrational progression assigned to an in‐plane deformation mode of the aromatic ring was observed for all three of them.^1^

Here we extend our work further and investigate the high‐temperature chemistry of the picolyl radicals, as generated by pyrolysis of the aminomethylpyridines **1**, **2** and **3** (see Scheme 1). Since secondary dissociation of picolyl leads to the formation of nitrogen‐containing open shell molecules, these reactions are relevant in the combustion of conventional fuels^2^ and bio‐fuels,^3^ in which substituted pyrroles or pyridines^4^ often occur as impurities. Therefore, their reaction products play an important role as reactive intermediates. While the products of the structurally related benzyl radical,^5^ which is a branching point in the decomposition of toluene,^6^ were studied in detail, information on the N‐heterocyclic analogues are sparse. Only the thermal reactions of 2‐picolyl have been investigated in shock tube experiments.^4^, ^7^ Acetylene and HCN were observed as major products, with cyclopentadiene and 1‐cyanocyclopentadiene being present in smaller amounts.^4^ Additionally, in astrochemistry and astrobiology reactive N‐containing heterocycles^8^ represent the first step in the growth of N‐containing polycyclic aromatic hydrocarbons (PANHs) in interstellar space and are incorporated in models to characterize the atmospheric chemistry of Titan, the biggest moon of Saturn.^9^ Here photoelectron photoion coincidence (PEPICO) spectroscopy^10^ and mass‐selective threshold photoelectron spectroscopy (ms‐TPES) are used to characterize the reaction products of aminomethylpyridines formed in a high temperature pyrolysis reactor. In these techniques the detection of ions and electrons is correlated, which permits to distinguish isomers of the same mass by their ionization energies and by the vibrational structure visible in the photoelectron spectrum. Thus, contributions from the pyrolysis of the precursor and reaction products with a different mass can be separated in the spectra. This has been shown in a number of studies on xylyl,^11^ xylylenes,^12^ butynyl radicals^13^ and ketenimine.^14^ Such isomer‐specific results deliver data for studies on catalytic reactors,^15^ flames^16^ and in kinetic experiments,^17^ where PEPICO is now applied as a tool for analyzing chemical reactions.

## Results and Discussion

### Mass spectra of the picolyl radical decomposition

**Figure 1:**
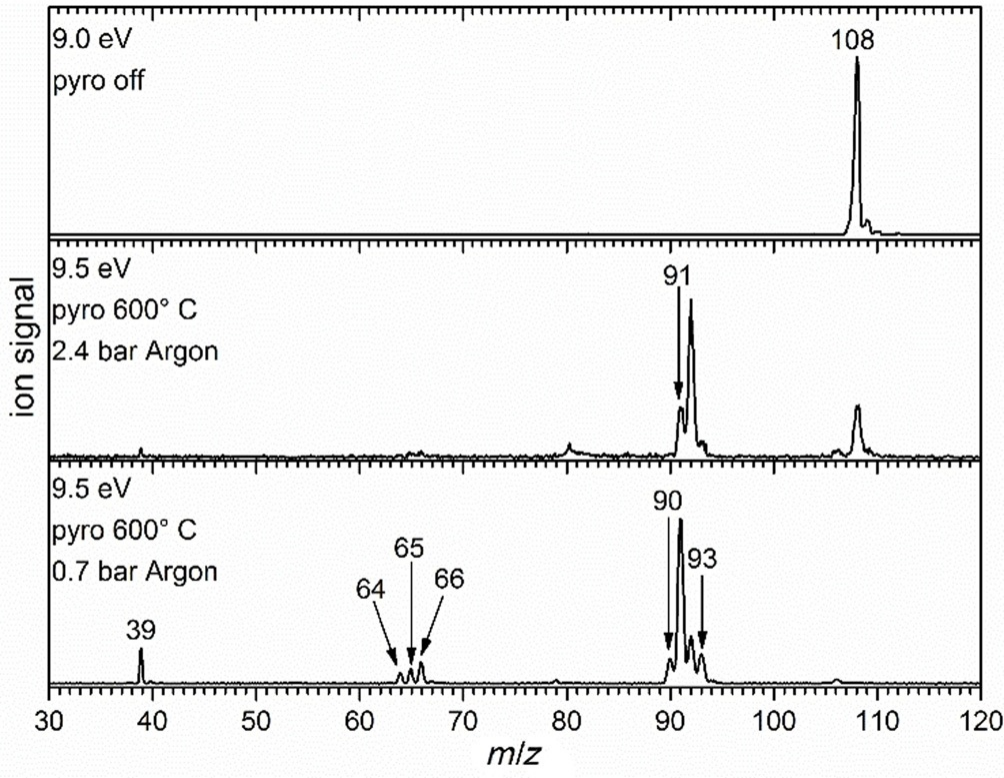
Mass spectra exemplary for 3‐aminomethylpyridine **2** at room temperature (top) and with 600° C pyrolysis temperature for 2.4 bar (center) and 0.7 bar Ar backing pressure (bottom).

Figure 1 illustrates mass spectra of 3‐aminomethylpyridine **2** with and without pyrolysis. For 2‐aminomethylpyridine **1** and 4‐aminomethylpyridine **3** similar spectra were recorded, which are therefore only given in the Supporting Information, Figure S1 and S2. In the spectrum recorded at 9.0 eV and without pyrolysis (top trace) only the precursor at *m*/*z*=108 is visible, accompanied by its ^13^C isotopologue as evident from the relative peak intensities. No fragments from dissociative photoionization are observed, because the onset is at significantly higher photon energies.

The spectra in the center and the bottom trace were recorded with active pyrolysis at two different backing pressures at a photon energy of 9.5 eV. The higher photon energy was chosen to detect all pyrolysis products. In both spectra, a fragmentation of the precursor **2** at 600 °C pyrolysis temperature is visible. A backing pressure of 2.4 bar of Argon, which corresponds to a higher dilution of the precursor (center trace), leads to an intense signal at *m*/*z*=92, which corresponds to the 3‐picolyl radical **5**. Precursor conversion is significant, but not complete. In addition, a further small signal appears at *m*/*z* = 91, corresponding to H‐atom loss from picolyl. When the backing pressure is reduced to 0.7 bar of Argon (bottom panel) corresponding to lower dilution of the precursor, a rich chemistry sets in, and several peaks appear with significant intensity that are hardly apparent with higher backing pressure. Due to the lower dilution in the jet and the increased residence time in the hot reactor, radical fragmentation and bimolecular reactions are stimulated. Consequently, the precursor is almost fully converted and the picolyl signal at *m*/*z*=92 is decreased. The signal at *m*/*z*=91 (H loss) increases significantly and peaks at *m*/*z*=90 (H_2_ loss) and 93 (H addition) are apparent. Additional mass peaks appear at *m*/*z*=64, 65 and 66, corresponding to loss of H_2_CN, HCN and CN from picolyl. Finally, a signal arises at *m*/*z*=39. Based on the mass spectra, a low backing pressure as in the bottom trace was chosen for further studies of the thermal reactions.

### ms‐TPE spectra of the intermediates and products

**Figure 2:**
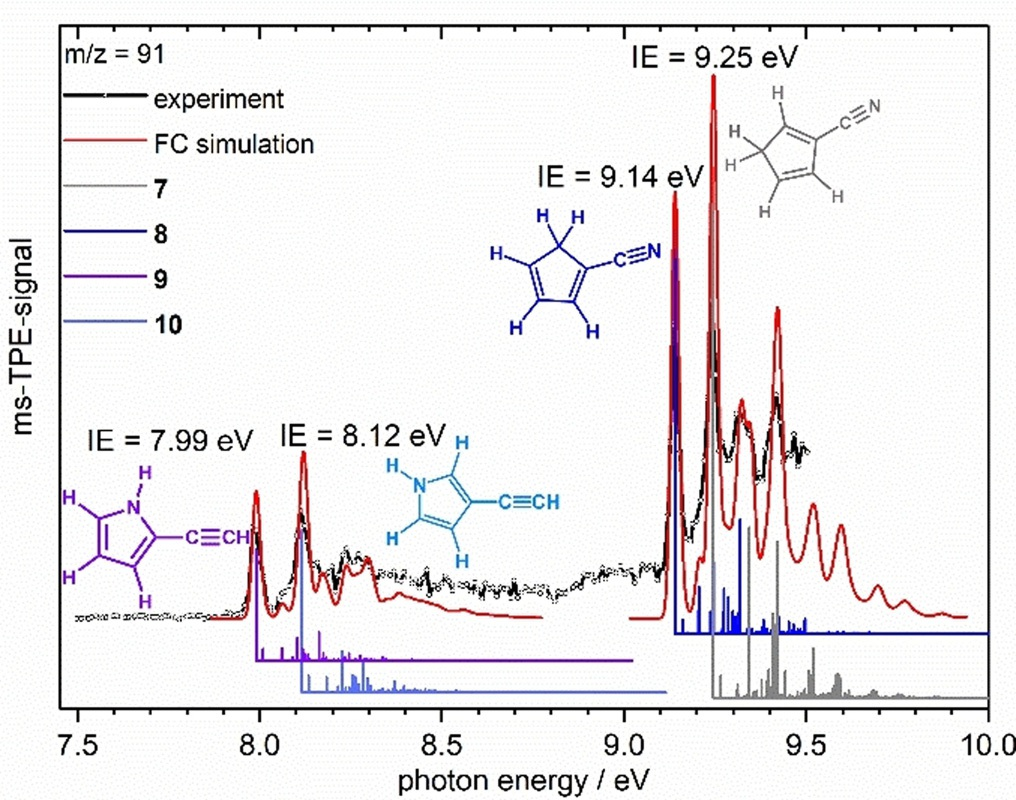
Mass‐selected TPE spectrum of *m*/*z*=91 and Franck–Condon simulations (red lines) for the four isomers: cyclopenta‐1,4‐diene‐1‐carbonitril **7** (grey sticks), cyclopenta‐1,3‐diene‐1‐carbonitril **8** (dark blue sticks), 2‐ethynyl‐1*H*‐pyrrole **9** (purple sticks) and 3‐ethynyl‐1*H*‐pyrrole **10** (light blue sticks) agree very well. The IE_ad_ indicated in the Figure were extracted for the various isomers.

In the following section the identification of the various mass peaks in the bottom trace of Figure 1 by ms‐TPES will be described. Unless otherwise noted the spectra below were recorded using precursor **2** and thus constitute products of 3‐picolyl **5**. However, very similar spectra were obtained using precursors **1** and **3**. The peak at *m*/*z*=93 is larger than expected for a ^13^C isotopologue and corresponds to the methylpyridine formed by H‐atom addition to **4**, **5** and **6**, as concluded from the ms‐TPE spectra. Since these products are stable and well‐studied closed‐shell molecules, the spectra are only shown in Figures S3–S5. The threshold photoelectron spectrum of *m*/*z*=91 after hydrogen loss of **5**, is given in Figure 2, spectra for all precursors are shown in Figure S7. The TPES was recorded from 7.50 eV to 9.50 eV with a step size of 5 meV, data were averaged for 120 s per data point.

The spectrum exhibits at least two band systems, separated by roughly 1 eV. Computations show that the most stable structures are associated with a ring contraction that yields two types of compounds, ethynyl pyrroles and cyanocyclopentadienes. Possible reaction pathways will be discussed below. The lower energy part of the spectrum from 7.90 to 8.50 eV can be assigned to the substituted pyrroles **9** and **10**. Based on the computed IE′s of 8.00 for **9** and 8.12 eV for **10** the major bands in this region at 7.99 and 8.12 eV were assigned to the adiabatic ionization energies IE_ad_ of the two compounds. The Franck Condon simulation for **9** and **10** (solid red line) also matches the less intense bands and is in excellent agreement with the experiment. By comparison with the computations the bands at 8.16 eV and 8.29 eV correspond to the in plane ring deformation of isomer **9** (+1441 cm^−1^) and **10** (+1415 cm^−1^) respectively, while the band at 8.20 eV was assigned to the stretching mode between the C atom of the ring and the *exo*‐ethynyl group of isomer **10** (+570 cm^−1^). A ring deformation mode of **10** including the *exo*‐ethynyl group (+930 cm^−1^) is responsible for the peak at 8.24 eV.

In the higher energy part of the spectrum from 9.00 to 9.50 eV the cyano substituted cyclopentadiene isomers **7** and **8** are observed. The two major bands at 9.14 and 9.25 eV represent the IE_ad_ of **8** and **7**, in excellent agreement with the calculated IE′s of 9.13 and 9.27 eV. Further vibrational bands are visible at 9.32 eV (with a recognizable shoulder at 9.34 eV) and at 9.42 eV. The symmetric and antisymmetric C=C stretching modes (see Supporting Information for description) in the ring, computed at 1463 and 1490 cm^−1^ for **8** are responsible for the band at 9.32 eV. The band at 9.42 eV represents the same two modes in **7**, computed at 1459 and 1504 cm^−1^. The shoulder at 9.34 eV is assigned to a ring deformation mode of **7** including the CH_2_ group (+822 cm^−1^).

**Figure 3:**
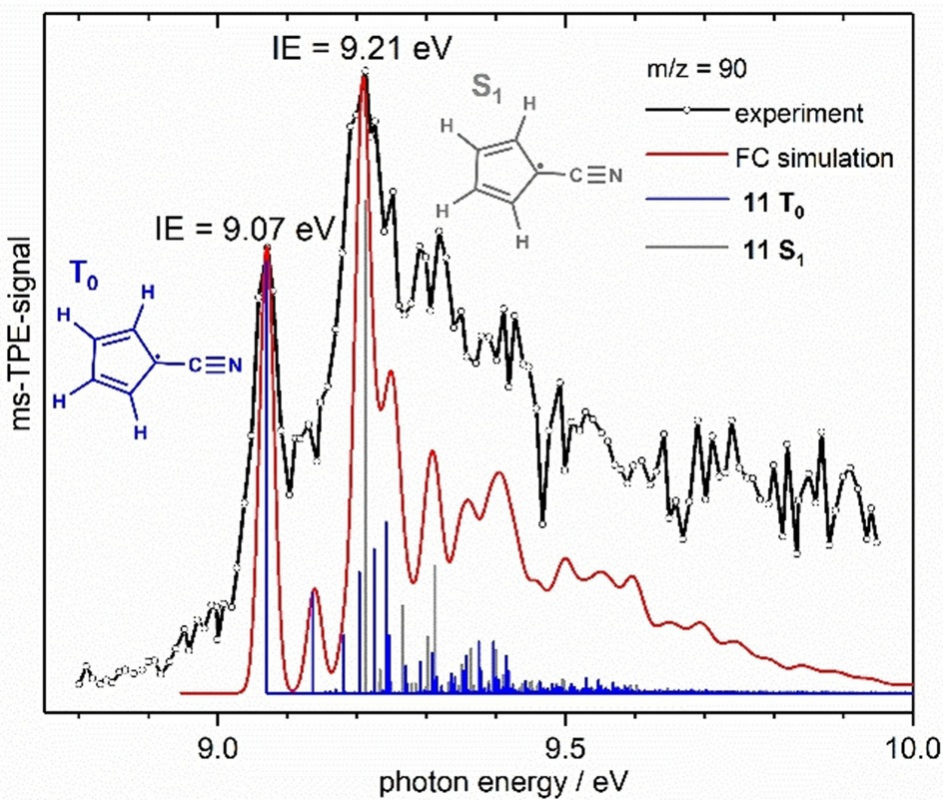
Mass‐selected TPE spectrum of *m*/*z*=90, obtained upon pyrolysis of **3** and a Franck‐Condon simulation for the cyanocyclopentadienyl radical **11**. The ionization energies for the two lowest cationic states are determined to be IE(*T*_0_)=9.07 eV and IE(S_1_)=9.21 eV.

In Figure 3 the broad ms‐TPE spectrum of *m*/*z*=90 (obtained upon pyrolysis of **3**) is displayed (black line with open circles). The mass formally corresponds to the consecutive loss of two H‐atoms starting from mass 92 or to H_2_ loss. The CBS‐QB3 computations indicate for cyanocyclopentadienyl **11** a triplet ground state for the cation and yield an IE of 9.11 eV. This is in very good agreement with the first major band in the spectrum at 9.07 eV. The present results revise the previous IE′s for **11** obtained from electron impact mass spectra (9.44 eV) and two‐photon ionization (9.05 eV).^18^ A second broader, but stronger transition starting at 9.21 eV is assigned to the first excited singlet state of the cation, S_1_. However, this value is significantly lower than the computational one of 9.36 eV. Nevertheless, the Franck Condon simulation for **11**, given in red, confirms that the broad band can be described by transitions into both the *T*_0_ and S_1_ states of the cation. Further bands appear at 9.13 and 9.25 eV and can tentatively be assigned with the aid of computations, the first one at 9.13 eV to an in‐plane ring deformation mode along the CN‐axis, computed at +556 cm^−1^, the second one at 9.25 eV to a symmetric CC stretch (+1436 cm^−1^), both in *T*_0_. A band at 9.30 eV however might correspond to a ring distortion in the S_1_ state (+822 cm^−1^).

For the radicals resulting from an H loss of the ethynyl pyrrole isomers **9** and **10** IE′s of 8.64 and 8.81 eV have been calculated, thus they are not expected to appear in the spectrum depicted in Figure 3, which starts at 8.8 eV only. A lower resolution survey spectrum from 7.5 to 9.5 eV, presented in Figure S6 (obtained upon pyrolysis of **2**), does not show evidence for the presence of further isomers of *m*/*z*=90. On the other hand, a further argument for the preferred formation of **11** is its comparably high stability. The cyanocyclopentadienyl radical is calculated to be 125.5 kJ mol^−1^ more stable than a possible radical resulting from **9** and 130.8 kJ mol^−1^ more stable than one resulting from **10**. Note that from both **7** and **8**, radical **11** is formed upon H‐loss, because charge and unpaired electron are resonantly stabilized.

**Figure 4:**
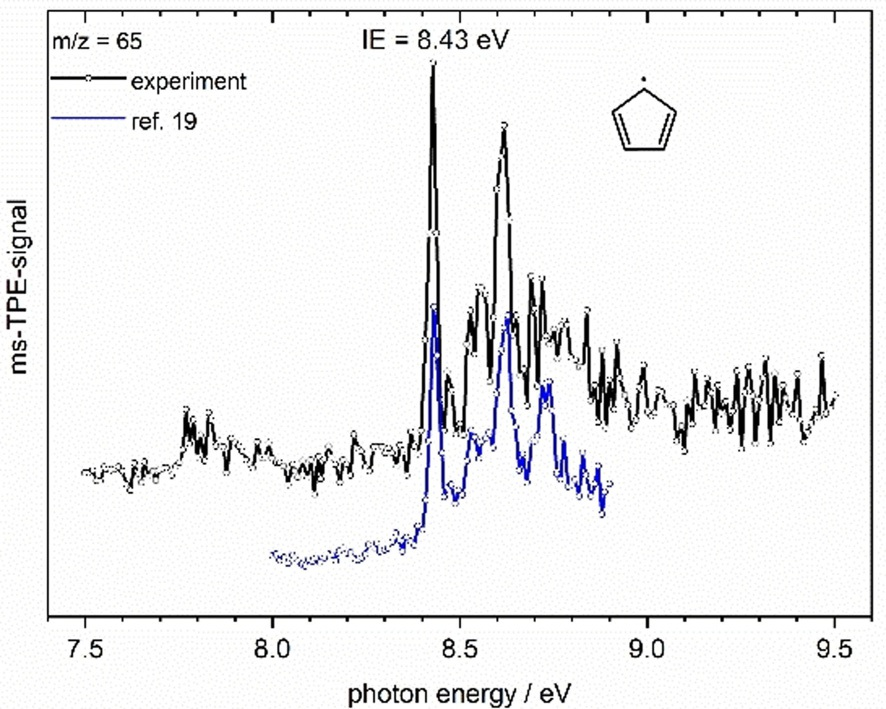
A comparison of the ms‐TPE spectrum of *m*/*z*=65 with a published spectrum of the cyclopentadienyl radical **12** (blue line) shows that **12** is formed in our experiment.^19^.

The ms‐TPE spectrum corresponding to the peak at *m*/*z*=65 (cf. Figure 1) is depicted in Figure 4 and assigned by comparison with previous spectra to the cyclopentadienyl radical **12**.^19^ It is most likely formed by HCN loss from picolyl (*m*/*z*=92) at higher temperatures, the possible reaction pathway is discussed below. The first band in the spectrum represents the adiabatic ionization energy of 8.43 eV and corresponds to an ionization into the *T*_0_ ground state of the cation. This value matches the one of 67969.2±4 cm^−1^ (≈8.427 eV) obtained from a zero kinetic energy photoelectron spectrum for the $\tilde{X}{{}^{2}E}_{1}^{{}^{'}{}^{'}}\to \tilde{X}{{}^{3}A}_{2}^{{}^{'}}$ transition by Wörner and Merkt.^20^ CBS‐QB3 computations yield a value of 8.47 eV for IE_ad_. Due to the Jahn–Teller distortion of the *D*_5*h*_ symmetric cyclopendienyl radical **12** a FC simulation is difficult and is not the main focus of this study.

The small features between 7.7 and 7.9 eV in Figure 4 originate possibly from the open chain isomer 1‐ethynylallyl radical. An IE of 7.88 eV has been calculated, but the signal is too small for a detailed analysis.

**Figure 5:**
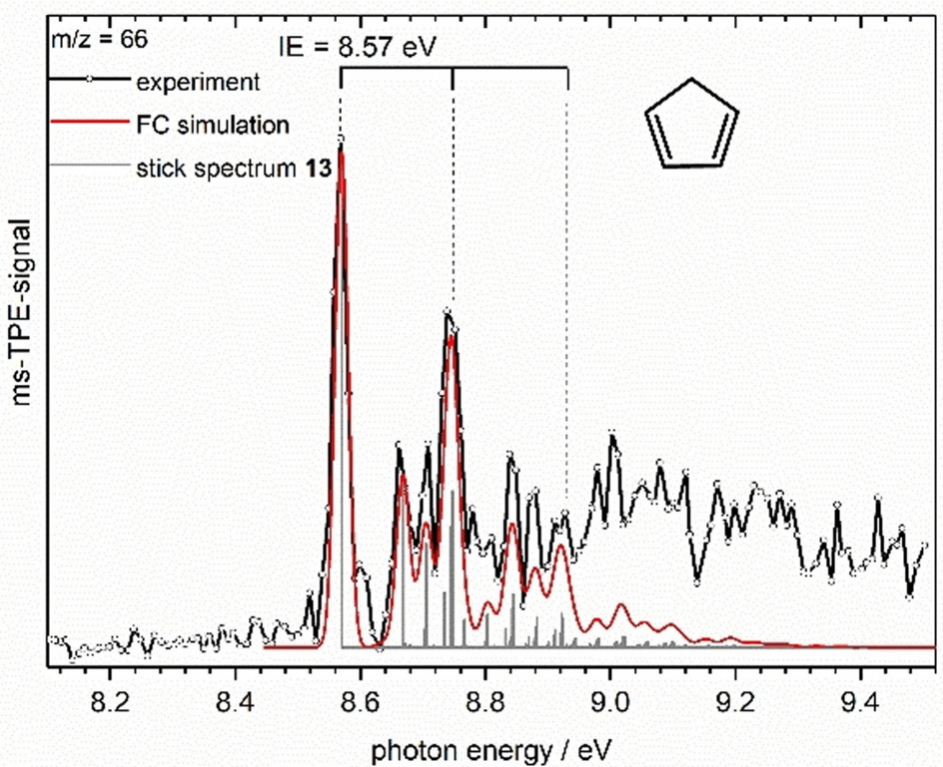
Mass‐selected TPE spectrum of *m*/*z*=66. The first major peak at 8.57 eV is assigned to the IE_ad_. For comparison, the Franck–Condon simulation for cyclopentadiene **13** is given.

The ms‐TPE spectrum of *m*/*z* = 66, shown in Figure 5 is dominated by the strong transition at 8.57 eV, which agrees well with the known IE_ad_ of cyclopentadiene **13**.^21^ The ionic ground state is due to ionization from the 1a_2_ π ‐orbital. Also, the Franck‐Condon simulation for cyclopentadiene matches the spectrum well. Several vibrational transitions are assigned by comparison with the earlier conventional photoelectron spectrum^22^ and computations. The first two are observed at 8.67 eV (ring distortion at +817 cm^−1^) and 8.71 eV (scissoring mode at +1128 cm^−1^). Two bands at 8.75 eV and 8.93 eV form two members of a vibrational progression of +0.18 eV that are assigned to the fundamental and first overtone of the symmetric C=C stretching modes (+1456 and 1478 cm^−1^). The bands at 8.85 and 8.88 eV represent combination bands. Cyclopentadiene **13** originates most likely from a hydrogen addition to *m*/*z*=65. The heat of reaction for the H‐addition to **12** is −80.8±1 kJ mol^−1^,^23^ derived from the computed value for Δ_r_ *H°* (c‐C_5_H_6_→c‐C_5_H_5_+H). The pyrrolyl radical, C_4_H_4_N represents a second species with *m*/*z*=66, which might be formed by thermal dissociation of ethynyl pyrrols. IE′s of 9.11 eV (S_0_) and 9.43 eV (T_1_) have been determined by TPES.^24^ This is in the part of the spectrum where the intensities deviate from the simulation. However, the TPE‐spectrum of pyrrolyl is furthermore characterized by a pronounced vibrational progression with a spacing of roughly 0.1 eV, which is not apparent in Figure 5. We therefore believe that the deviations are due to the limited accuracy of FC simulations or autoionizing transitions and conclude that pyrrolyl may only be present in small amounts.

**Figure 6:**
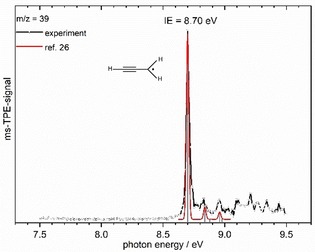
Mass‐selected TPE spectrum of *m*/*z*=39. A Franck–Condon simulation for the propargyl radical **14** (red line) based on the calculations of Botschwina et al. shows an excellent agreement.^26^

The ms‐TPES of *m*/*z*=39 shown in Figure 6 is assigned to the propargyl radical **14**. Scheer et al computed Δ_r_ *H*° (c‐C_5_H_5_→HCCH+HCCCH_2_)=310±13 kJ mol^−1^ for the acetylene loss from the cyclopentadienyl radical **12**.^25^ Given the observation of **12**, this reaction provides therefore a convincing explanation for the formation of **14** in our experiments.

The strong band at 8.70 eV corresponds to the adiabatic ionization energy and is in perfect agreement with previous theoretical^26^, ^27^ and experimental values.^28^, ^29^ For comparison the simulation (red line) based on the Franck–Condon (FC) factors calculated by Botschwina and Oswald is given,^26^ which exhibits an excellent agreement with the experimental data. The two small features at 8.83 and 8.96 eV that originate from the pseudosymmetric and pseudoantisymmetric CC stretching vibration are also well represented.

**Figure 7:**
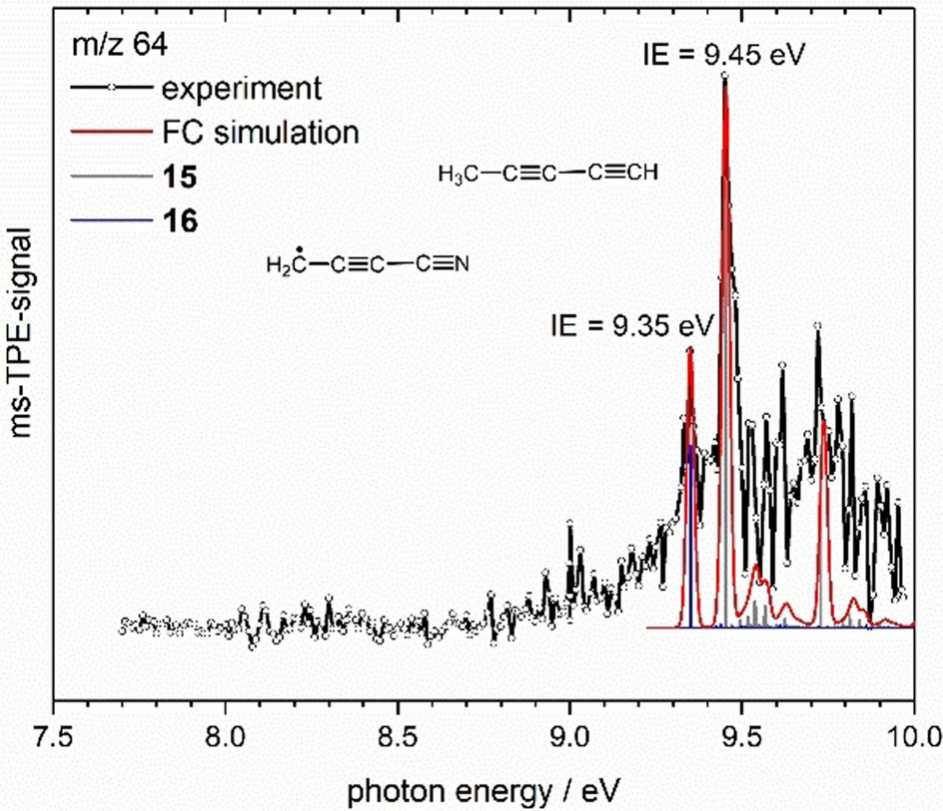
Comparison of the ms‐TPE spectrum of *m*/*z*=64 (obtained from **3**) with a Franck‐Condon simulation (red line) for the cyanopropynyl radical **16** (blue sticks) and penta‐1,3‐diyne **15** (grey sticks).

Figure 7 displays the ms‐TPES of *m*/*z*=64, obtained from pyrolysis of **3**. The experimental data were recorded in 10 meV steps. Using precursor **2** a similar spectrum was obtained, but the scan was stopped at 9.45 eV. The broad band is comprised by ionization from two different molecules, penta‐1,3‐diyne **15** (grey sticks) and cyanopropynyl **16** (blue sticks). The strongest transition at 9.45 eV was assigned to the adiabatic ionization energy of **15**, which agrees well with the value of 9.50±0.02 eV measured by Maier for **15**.^30^ The first peak at 9.35 eV was correlated with the adiabatic ionization energy of **16** and matches our CBS‐QB3 value of 9.37 eV. Further peaks are apparent at 9.62 and 9.72 eV and are assigned to the methyl bending mode (+1430 cm^−1^) and the symmetric stretch of the two C≡C triple bonds (+2291 cm^−1^) in penta‐1,3‐diyne **15**. Overall the agreement of the FC simulation with the experimental data is good, given that two transitions overlap and the signal/noise ratio is low.

In Table 1 all IE′s determined in the present work are summarized and compared with computations and previous work, when available. As visible, for some species no IE_ad_ has been previously reported, while for other species earlier values are revised. A very good agreement with computations is achieved.

**Table 1:** Ionization energies (IEs) determined in the present work. Experimental values are generally accurate within ±0.02 eV. IE_comp_ calculated with CBS‐QB3 are given for comparison.

Molecule	IE_exp_ [eV]	IE_comp_ [eV]	IE_lit_ [eV]
2‐picolyl **4**	7.70	7.73	7.70±0.02^1^
3‐picolyl **5**	7.59	7.65	7.59±0.01^1^
4‐picolyl **6**	8.01	8.06	8.01±0.01^1^
cyclopenta‐1,4‐diene‐1‐carbonitrile **7**	9.25	9.27	–
cyclopenta‐1,3‐diene‐1‐carbonitrile **8**	9.14	9.13	9.70^31^
2‐ethynyl‐1*H*‐pyrrole **9**	7.99	8.00	–
3‐ethynyl‐1*H*‐pyrrole **10**	8.12	8.12	–
cyanocyclopentadienyl radical **11**(T_0_)	9.07	9.11	9.05±0.0218b
cyanocyclopentadienyl radical **11**(S_1_)	9.21	9.36	–
cyclopentadienyl radical **12**	8.43	8.47	8.4271^20^
cyclopentadiene **13**	8.57	8.60	8.5321a
propargyl radical **14**	8.70	8.74	8.7005^29^
penta‐1,3‐diyne **15**	9.45	9.47	9.50±0.02^30^
cyanopropenyl radical **16**	9.35	9.37	–

### Decomposition mechanism of the 2‐picolyl radical 4

**Scheme 2:**
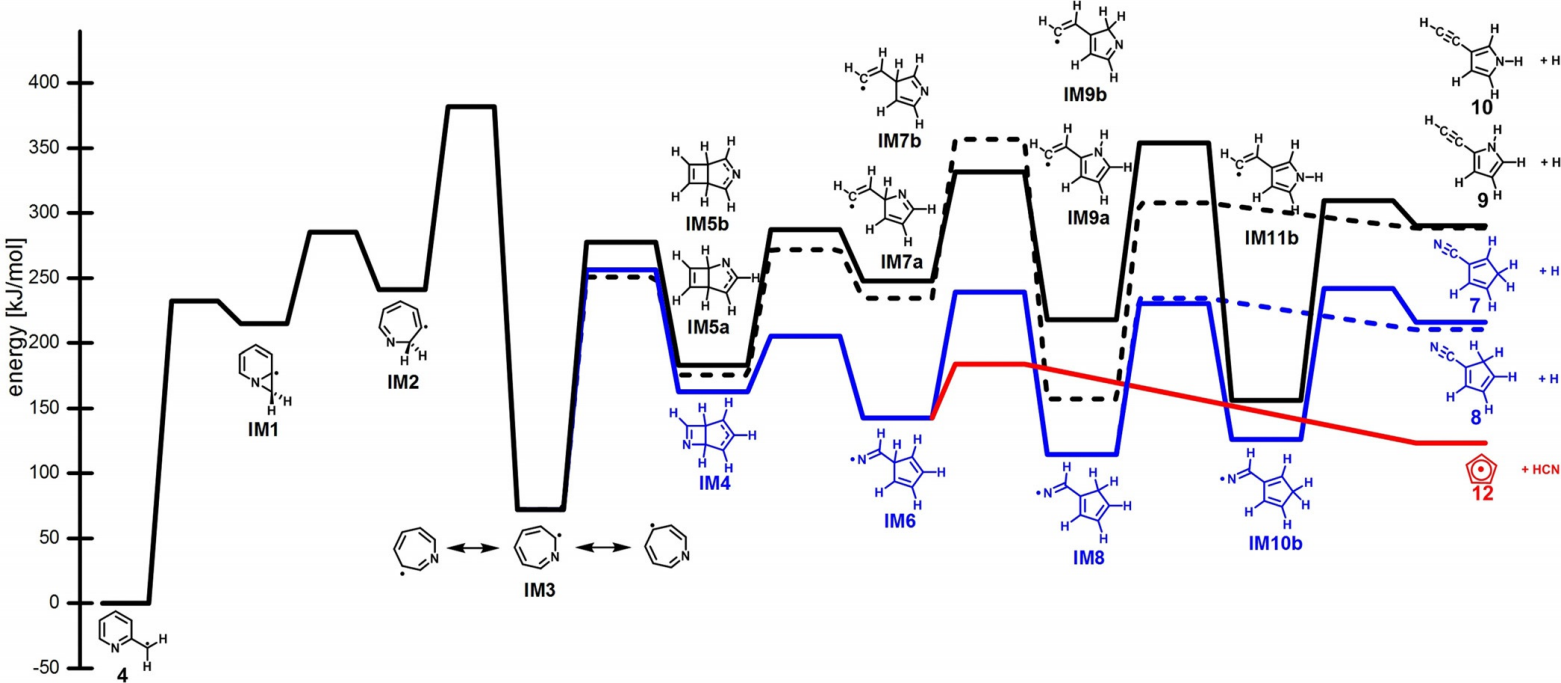
First steps in the decomposition of the picolyl isomer **4**, computed by CBS‐QB3. 2‐picolyl **4** has been set to zero.

In Scheme 2 the mechanism for the first dissociation steps of the picolyl radical **4** are depicted, while the energies of the stationary points are summarized in Table S8. The energies for the initial H‐atom loss were computed to rationalize the formation of the four isomers detected in the ms‐TPE spectra, **7** (blue line), **8** (blue dashed line), **9** (black dashed line) and **10** (black line) from the three picolyl radicals. In addition, the HCN loss from picolyl to cyclopentadienyl radical **12** is represented as a red line. For all picolyl isomers dissociation proceeds via the 7‐membered ring **IM3**, thus the subsequent steps are identical. The first steps leading to **IM3** in Scheme 2 are given for isomer **4**, but for the other isomers **5** and **6** only slightly different barriers were obtained for the steps to **IM3**. Therefore, they are only given in Figure S8.

The first intermediate **IM1** in the dissociation of **4** is the bicycle **IM1**, which is +215 kJ mol^−1^ higher in energy due to ring strain. It is followed by electrocyclization to the seven‐membered ring **IM2**, which is then stabilized in a 1,3 H‐shift, yielding the resonance stabilized azepinyl radical **IM3**. This is +72 kJ mol^−1^ less stable than reactant **4** and resembles the cycloheptatrienyl radical, a key intermediate in the decomposition of benzyl.^32^ **IM3** can then cyclize to three bicyclic structures with the N atom either in the four (**IM4**) or five membered ring (**IM5a** and **b**).

All three pathways proceed over barriers with similar energies, **IM5a** is only 12 kJ mol^−1^ and **IM5b** 20 kJ mol^−1^ less stable than **IM4**. While reaction via **IM4** proceeds to cyclopentadiene carbonitriles **7** and **8**, reaction via **IM5** finally leads to the ethynyl pyrroles **9** and **10**. In all pathways the four membered ring opens to form either a substituted pyrrole with the nitrogen in one‐ (**IM7a**) or two‐position (**IM7b**) to the vinyl substituent or a cyano‐substituted cyclopentadiene (**IM6**). In the next step a 1,2 H‐migration forms the intermediates **IM8**, **IM9a** and **IM9b**. For **IM8** and **IM9b** this step is exothermic by around 30 kJ mol^−1^, for **IM9a** even by 77 kJ mol^−1^. Alternatively, **IM6** can dissociate over a low‐energy barrier to HCN and the resonance‐stabilized cyclopentadienyl radical **12** (red line). While **12** has been observed by ms‐TPES, the ionization energy of HCN of 13.60 eV is too high for a detection in the photon energy range of the experiment. We note that **12** might alternatively be formed by CN‐loss from **7** and **8**, but due to the low barrier the direct pathway from picolyl will be an efficient route to **12**. Note that the mechanism of the subsequent unimolecular dissociation of **12** to C_3_H_3_+C_2_H_2_ has been described in detail before.^25^ Products **8** and **9** can be formed directly by H‐atom loss from **IM8** and **IM9a** (dashed lines), while for **IM9b** a second 1,2 H shift to **IM11b** was computed before product **10** is formed (black line). For **IM8** a second pathway over a second consecutive 1,2 H shift similar to **IM11b** is possible (blue line). Similarly, H‐atom loss to product **7** proceeds from **IM8** via **IM10b**. All four products **7**, **8**, **9** and **10** were assigned in the ms‐TPE spectra, suggesting that all pathways indicated in Scheme 2 are active. Assuming comparable ionization cross sections for all products, the bigger signal observed for **7** and **8** (blue lines) compared to **9** and **10** might be explained by the more favorable reaction energetics. From the chemical structures it is evident that a further H‐atom loss to the experimentally observed cyanocyclopentadienyl radical **11** is feasible from **7** and **8**. For **9** and **10** no low‐lying H‐atom loss channel is apparent and possible products are not observed experimentally. An alternative route to **11** would be H_2_ loss from picolyl, but no obvious reaction pathway has been found.

Note that a similar mechanism for decomposition of **4** has already been suggested by Doughty and Mackie, although in less detail.^4^ However they concluded that “The resonance stabilized seven‐membered ring intermediate… appears unlikely” because “one would expect to observe both pyrrole and substituted pyrroles, in the reaction products”. In their shock tube experiments only acetylene, HCN, cyclopentadiene and 1‐cyanocyclopentadiene were observed. The detection of ethynyl pyrroles by ms‐TPES in the present work thus provides strong evidence for the mechanism outlined in Scheme 2 and for a reaction that proceeds via intermediate **IM3**.

Finally, it is of interest to compare the unimolecular reactions of picolyl with the reactions of the related benzyl radical. In benzyl, two dominant pathways were identified,^33^ (a) H‐atom loss to fulvenallene and (b) reaction to c‐C_5_H_5_+C_2_H_2_. The equivalent to (b), reaction to c‐C_5_H_5_+HCN is also observed here for all picolyl isomers. However, the ms‐TPES in Figure 2 rules out a large contribution of N‐heterocyclic analogues of (a) and ethynyl pyrroles are formed from picolyl, instead. We have calculated the reaction pathway of 2‐picolyl to aza‐fulvenallene (see Table S7 and Figure S10) and found that it possesses a similar rate‐limiting barrier as the formation of **IM3**, but the products are less stable than **7**, **8** and **9**. Like in the benzyl radical, where fulvenallene is observed upon hydrogen atom loss, aza‐fulvenallenes could in principle appear as reaction products of picolyl. However, this is in contrast to our experimental findings. Although energetically accessible, we did not detect aza‐fulvenallene species (computed IE′s are 8.47 and 9.21 eV), which indicate either a rapid fragmentation to products with higher IE (CN, HCN, acetylene) or very low concentrations below the detection limit, due to the lower stability as compared to **7**–**10**.

## Conclusion

In this study we have focused on the decomposition of 2‐picolyl **4**, 3‐picolyl **5** and 4‐picolyl **6** radicals in a hot pyrolysis reactor, which yielded a range of products **7**–**16**. All products were identified by mass‐selected threshold photoelectron spectroscopy. Identical products were observed for all three picolyl isomers. In a first step a hydrogen loss or addition takes place, resulting in mass 91 or 93, respectively. H‐atom addition yields the stable methylpyridines. In contrast, H‐atom loss leads to two different types of structures, ethynyl pyrroles and cyclopentadiene carbonitriles. Four product isomers were identified in the ms‐TPES by comparison with computations and Franck–Condon simulations: 2‐ethynyl‐1*H*‐pyrrole **9** (IE=7.99±0.02 eV) and 3‐ethynyl‐1*H*‐pyrrole **10** (IE=8.12±0.02 eV) as well as cyclopenta‐1,4‐diene‐1‐carbonitril **7** (IE=9.25±0.02 eV) and cyclopenta‐1,3‐diene‐1‐carbonitril **8** (IE=9.14±0.02 eV). Only **8** has been observed before, however, the IE had to be revised considerably. A second consecutive hydrogen loss from **7** and **8** yields the cyanocyclopentadienyl radical **11**, which has an ionic triplet ground state. The ionization energy was determined to be 9.07±0.02 eV. In addition, the S_1_ state was observed and the ionization energy was characterized to be 9.21±0.02 eV. A product at *m*/*z*=65 was assigned to the cyclopentadienyl radical **12**. Most likely it is formed by HCN elimination from picolyl radicals. Product **12** has several opportunities for further reactions. While a further hydrogen loss yields penta‐1,3‐diyne **15**, H‐atom addition forms cyclopentadiene **13**, respectively. Alternatively, it decomposes to ethyne and the propargyl radical **14**.

Based on computations a mechanism for the dissociation of picolyl is proposed that proceeds via a seven‐membered ring intermediate. This intermediate resembles the cycloheptatrienyl radical, which is a key species in the high‐temperature reactions of benzyl. Assuming this pathway, the formation of all products can be explained. In a previous study the seven‐membered ring intermediate has been discarded, due to the absence of substituted pyrroles. The detection of **9** and **10** described above thus provides evidence for the computed reaction pathway. Since the reaction proceeds through the same intermediate **IM 3** from all picolyl isomers, the same reaction products and comparable ratios were observed from all precursors.

To summarize, the dissociation of picolyl radicals has been explored and a possible pathway for the first H‐atom loss has been outlined. Several products have been identified and characterized by photoelectron spectroscopy, yielding a wealth of structural information on a number of intermediates relevant for biofuel processing.

## Experimental Section

All experiments were performed at the Swiss Light Source (SLS) storage ring in Villigen, Switzerland. The details on both the x04db beamline^34^ and the i^2^PEPICO setup^35^ are available in the literature; thus, only a brief summary is given here. Vacuum ultraviolet (VUV) synchrotron radiation (SR) provided by a bending magnet and collimated onto a 150 lines mm^−1^ plane grating monochromator was used for ionization. The photon energy resolution *E*/Δ*E* is about 1.5×10^3^. Depending on the photon energy range either a MgF_2_ window (from 5 to 10 eV) or a rare gas filter operating with 10 mbar of a Kr/Ar/Ne mixture (from 7 to 14 eV) were employed to suppress higher harmonic radiation. For the experiments described in this work the photon energy was scanned in 5 or 10 meV steps and calibrated using the 11s${}^{'}$ –13s${}^{'}$ autoionization resonances of Ar in first and second order. All spectra are corrected for photon flux.

The liquid precursors were purchased from Sigma–Aldrich with a purity of 98 % or higher. Glass wool, soaked with the liquids, was positioned in an in‐vacuum sample container directly in front of the nozzle. Due to their low vapor pressure, the aminomethylpyridines **1**, **2** and **3** were heated between 30–40 °C to transfer them into the gas phase. They were seeded in Ar and expanded through a 100 μm nozzle into the pyrolysis reactor, an electrically heated silicon carbide (SiC) tube^36^ to generate the thermal products. The skimmed beam was overlapped with the SR. After ionization electrons and ions were extracted vertically in opposite direction. The i^2^PEPICO setup consists of two velocity map imaging (VMI) spectrometers for photoelectrons and photoions.17a, 35, 37 The cations and electrons were accelerated in an electric field and imaged on a position sensitive delay‐line anode (Roentdek DLD40). Cations and electrons from the same ionization event were correlated employing a multiple start/multiple stop data acquisition Scheme, permitting to record ion mass‐selected photoelectron spectra.^38^ False coincidences, for example, background or hot electrons, were subtracted following the procedure given in Ref. 39 Threshold photoelectrons were selected with a resolution of 3–5 meV.

All quantum chemical computations were performed with the Gaussian 09 suite of programs.^40^ The composite CBS‐QB3 method was used to calculate ionization energies and stationary points on the reaction coordinate.^41^ This method optimizes the molecular geometries and computes the vibrational wavenumbers and force constants of the neutral and ionic ground state. The ms‐TPE spectra of the decomposition products **7**–**16** were simulated either by a Franck‐Condon simulation at 0 K with the program FCfit version 2.8.20 or at 600–800 K using the program ezSpectrum.^42^

## Conflict of interest

The authors declare no conflict of interest.

## Supporting information

As a service to our authors and readers, this journal provides supporting information supplied by the authors. Such materials are peer reviewed and may be re‐organized for online delivery, but are not copy‐edited or typeset. Technical support issues arising from supporting information (other than missing files) should be addressed to the authors.

Supplementary
