# Supplementary material for: Decomposition of Picolyl Radicals at High Temperature: A Mass Selective Threshold Photoelectron Spectroscopy Study
Source: Chemistry. 2019 Dec 5;25(72):16652–9. doi: 10.1002/chem.201903937 (PMC6972682; doi:10.1002/chem.201903937)
Supplement: Supplementary file 1 — Supplementary [file CHEM-25-16652-s001.pdf]

CHEMISTRY
A European Journal

Supporting Information

Decomposition of Picolyl Radicals at High Temperature: A Mass Selective Threshold Photoelectron Spectroscopy Study

Engelbert Reusch,[a] Fabian Holzmeier,[b] Marius Gerlach,[a] Ingo Fischer,*[a] and Patrick Hemberger*[c]

chem_201903937_sm_miscellaneous_information.pdf

Table of Contents

a) Computed Geometries of **7**, **8**, **9**, **10** and **11** and their cations. S2

b) Mass spectra of 2-aminomethylpyridine **1** and 4-aminomethylpyridine **3** with and without pyrolysis. S8

c) Additional mass-selected threshold photoelectron spectra S10

d) Absolute energies of **9** and **10** in comparison with aza-fulvenallenes S15

e) Illustration of the vibrational modes of **7**, **8**, **9** and **10**. S16

f) Energies of stationary points along the reaction coordinate for the calculated decomposition pathway of 2-picolyl **4** shown in scheme 2 of the main paper. S17

g) Further computed reaction pathways. S19

a) Computed Geometries of 7, 8, 9, 10 and 11 and their cations.

Table S1. Geometry parameters of cyclopenta-1,4-diene-1-carbonitrile **7**, computed by DFT using CBS-QB3 (based on the B3LYP functional and a 6-311g (2d,d,p) basis set).

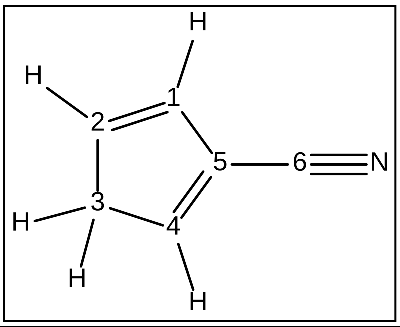

1. bond length (R) in Å			2. in-plane angle (A) in °		
	neutral	cation		neutral	cation
R(1,2)	1.34	1.39	A(1,2,3)	109.6	110.4
R(2,3)	1.50	1.49	A(2,3,4)	103.5	102.6
R(3,4)	1.50	1.49	A(3,4,5)	108.8	109.3
R(4,5)	1.35	1.41	A(4,5,1)	109.5	109.1
R(1,5)	1.48	1.42	A(5,1,2)	108.7	108.7
R(5,6)	1.42	1.41	A(5,6,N)	179.0	179.8
R(6,N)	1.16	1.16			

Table S2. Geometry parameters of cyclopenta-1,3-diene-1-carbonitrile **8**, computed by DFT using CBS-QB3 (based on the B3LYP functional and a 6-311g (2d,d,p) basis set).

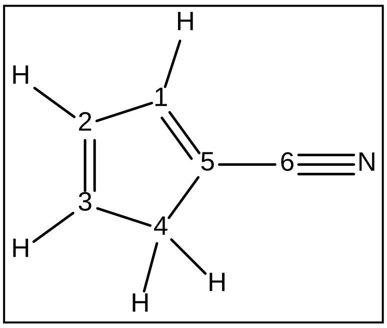

1. bond length (R) in Å			2. in-plane angle (A) in °		
	neutral	cation		neutral	cation
R(1,2)	1.46	1.41	A(1,2,3)	109.3	109.2
R(2,3)	1.35	1.39	A(2,3,4)	109.7	110.5
R(3,4)	1.50	1.49	A(3,4,5)	102.8	101.9
R(4,5)	1.51	1.51	A(4,5,1)	108.9	109.4
R(1,5)	1.36	1.41	A(5,1,2)	109.3	109.1
R(5,6)	1.41	1.39	A(5,6,N)	178.1	179.3
R(6,N)	1.16	1.16			

Table S3. Geometry parameters of 2‑ethynyl-1*H*-pyrrole **9**, computed by DFT using CBS-QB3 (based on the B3LYP functional and a 6-311g (2d,d,p) basis set).

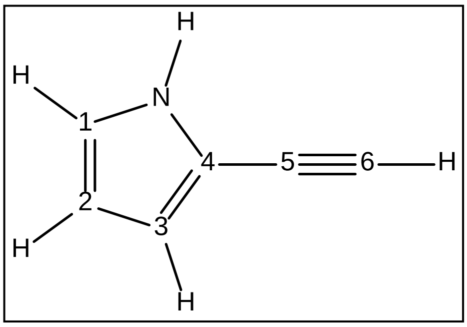

1. bond length (R) in Å			2. in-plane angle (A) in °		
	neutral	cation		neutral	cation
R(N,1)	1.37	1.35	A(N,1,2)	107.9	108.9
R(1,2)	1.38	1.43	A(1,2,3)	107.5	107.4
R(2,3)	1.42	1.37	A(2,3,4)	107.7	107.5
R(3,4)	1.39	1.44	A(3,4,N)	106.9	106.9
R(4,N)	1.38	1.39	A(4,N,1)	110.0	109.3
R(3,5)	1.41	1.38	A(3,5,6)	179.5	178.7
R(5,6)	1.21	1.22			

Table S4. Geometry parameters of 3-ethynyl-1*H*-pyrrole **10**, computed by DFT using CBS-QB3 (based on the B3LYP functional and a 6-311g (2d,d,p) basis set).

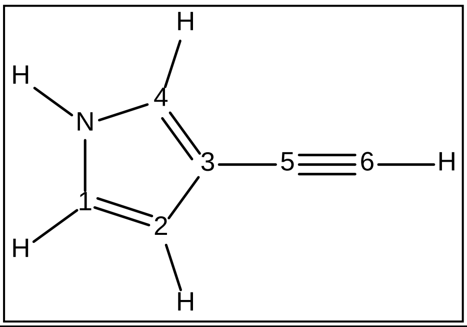

1. bond length (R) in Å			2. in-plane angle (A) in °		
	neutral	cation		neutral	cation
R(N,1)	1.38	1.41	A(N,1,2)	107.8	108.7
R(1,2)	1.37	1.37	A(1,2,3)	107.5	107.1
R(2,3)	1.43	1.42	A(2,3,4)	106.8	106.6
R(3,4)	1.39	1.45	A(3,4,N)	107.8	107.3
R(4,N)	1.37	1.32	A(4,N,1)	110.1	110.2
R(4,5)	1.42	1.38	A(4,5,6)	179.7	179.4
R(5,6)	1.21	1.22			

Table S5. Geometry parameters of cyanocyclopentadienyl radical **11** (**S_1**), computed by DFT using CBS-QB3 (based on the B3LYP functional and a 6-311g (2d,d,p) basis set).

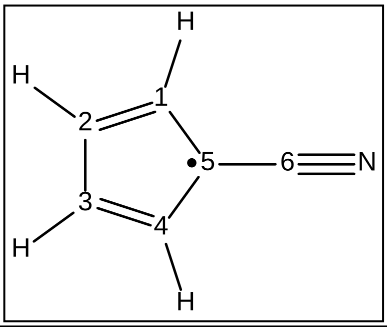

1. bond length (R) in Å			2. in-plane angle (A) in °		
	neutral	cation		neutral	cation
R(1,2)	1.36	1.42	A(1,2,3)	108.7	111.8
R(2,3)	1.48	1.47	A(2,3,4)	108.7	107.2
R(3,4)	1.36	1.34	A(3,4,5)	106.8	106.8
R(4,5)	1.45	1.57	A(4,5,1)	108.9	108.5
R(1,5)	1.45	1.37	A(5,1,2)	106.8	105.8
R(5,6)	1.40	1.39	A(5,6,N)	180.0	178.0
R(6,N)	1.16	1.16			

Table S6. Geometry parameters of cyanocyclopentadienyl radical **11 (T_0)**, computed by DFT using CBS-QB3 (based on the B3LYP functional and a 6-311g (2d,d,p) basis set).

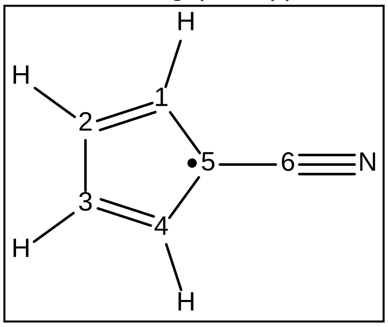

1. bond length (R) in Å			2. in-plane angle (A) in °		
	neutral	cation		neutral	cation
R(1,2)	1.36	1.42	A(1,2,3)	108.7	108.5
R(2,3)	1.48	1.42	A(2,3,4)	108.7	108.5
R(3,4)	1.36	1.42	A(3,4,5)	106.8	107.7
R(4,5)	1.45	1.44	A(4,5,1)	108.9	107.6
R(1,5)	1.45	1.44	A(5,1,2)	106.8	107.7
R(5,6)	1.40	1.40	A(5,6,N)	180.0	180.0
R(6,N)	1.16	1.16			

b) mass spectra of 2-aminomethylpyridine 1 and 4-aminomethylpyridine 3 with and without pyrolysis.

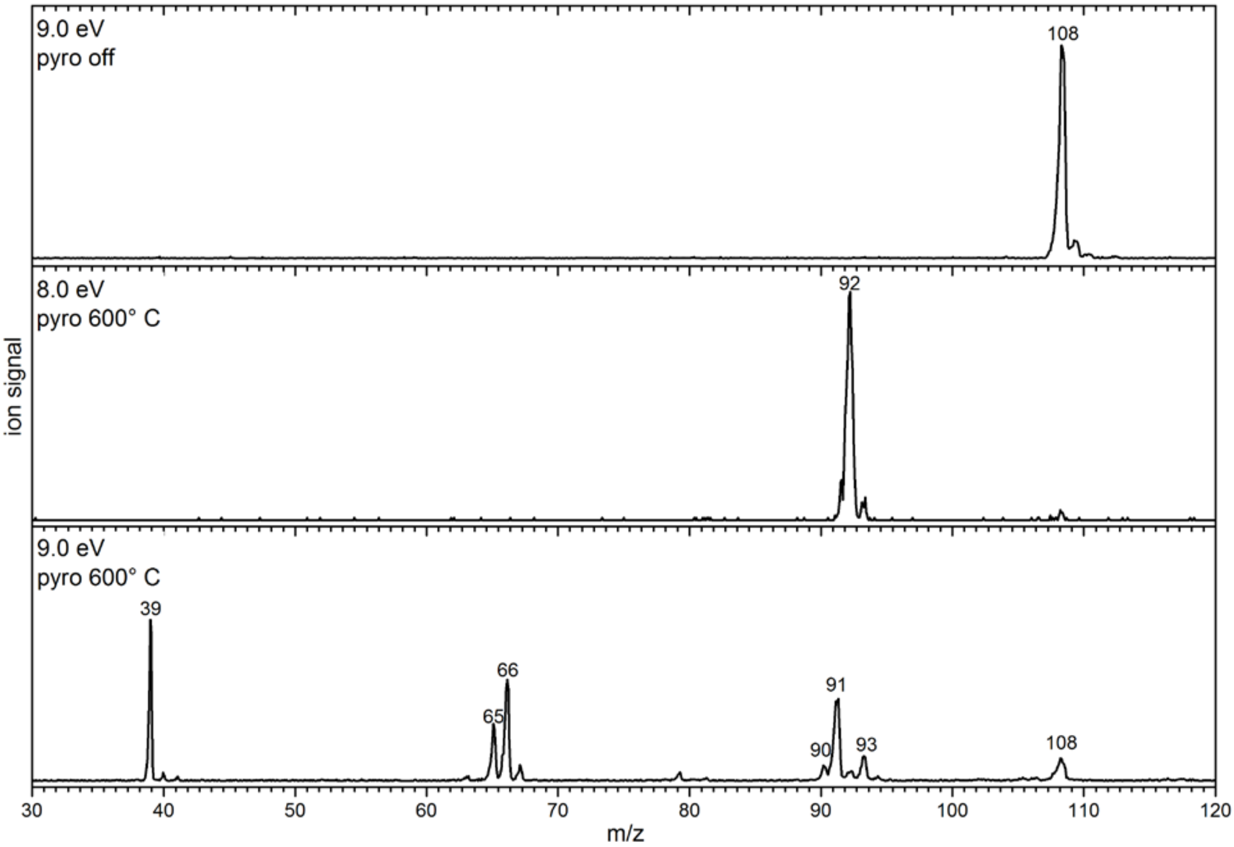

Figure S1. Mass spectra for 2-aminomethylpyridines **1** at room temperature (top) and with 600° C pyrolysis temperature at 8.0 eV (center) and at 9.0 eV (bottom).

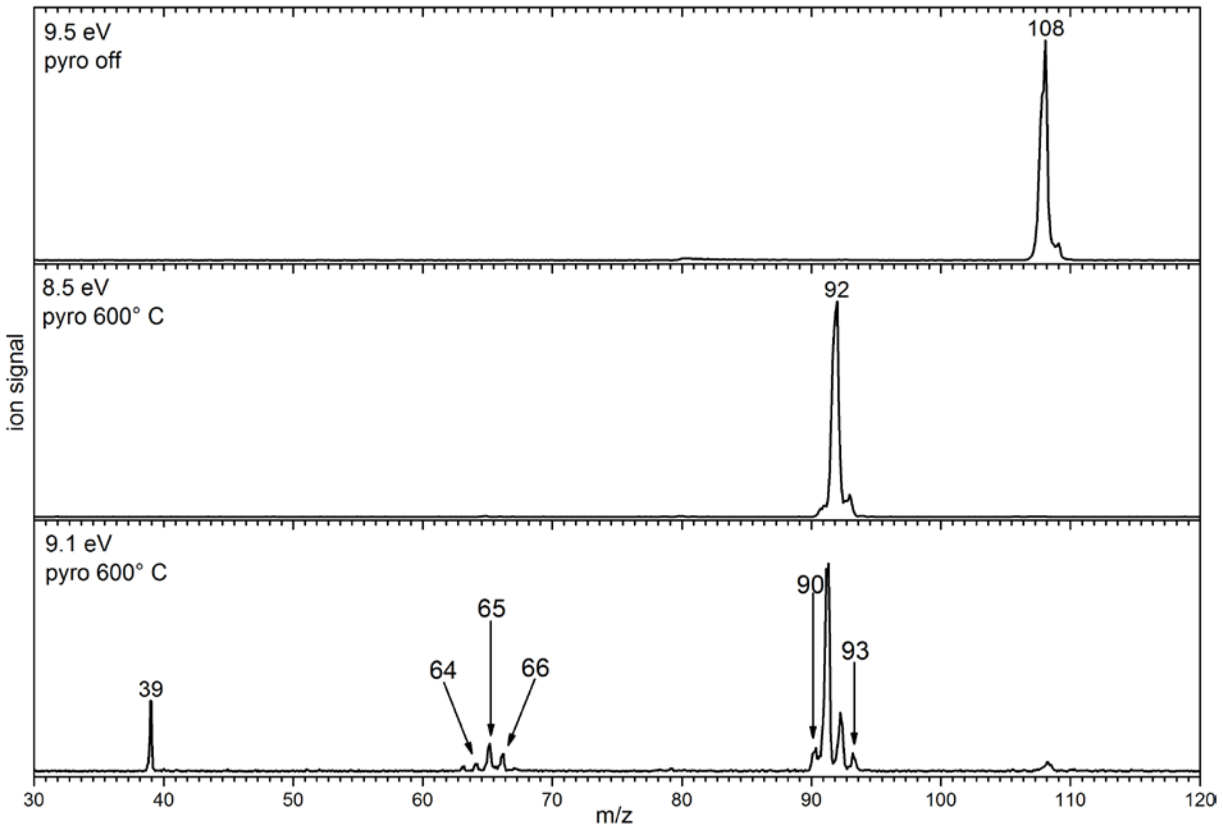

Figure S2. Mass spectra for 4-aminomethylpyridines **3** at room temperature (top) and with 600° C pyrolysis temperature at 8.5 eV (center) and at 9.1 eV (bottom).

c) Additional mass-selected threshold photoelectron spectra

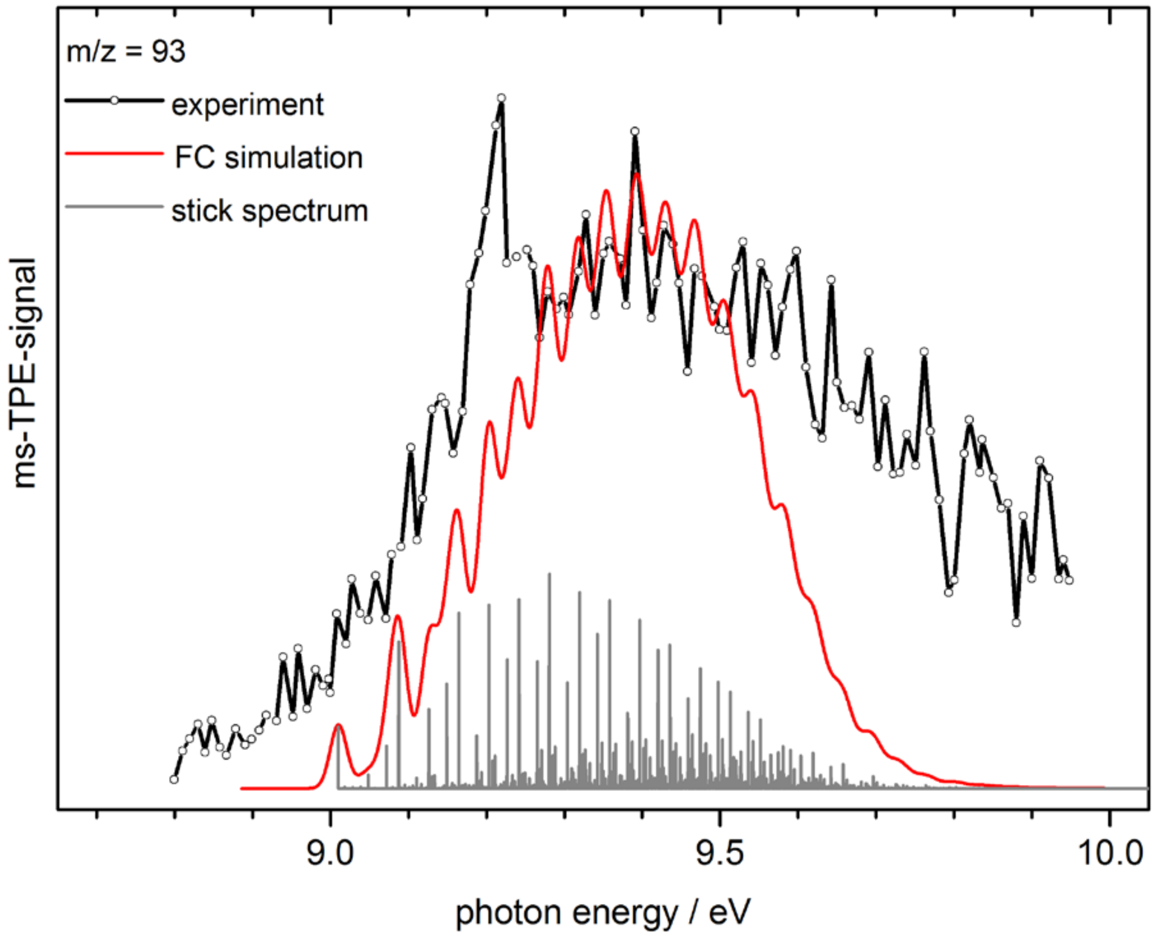

Figure S3. ms-TPE spectrum of *m*/*z* = 93 and a Franck-Condon simulation for 2-methylpyridine (red line) based on the CBS-QB3 level of theory.

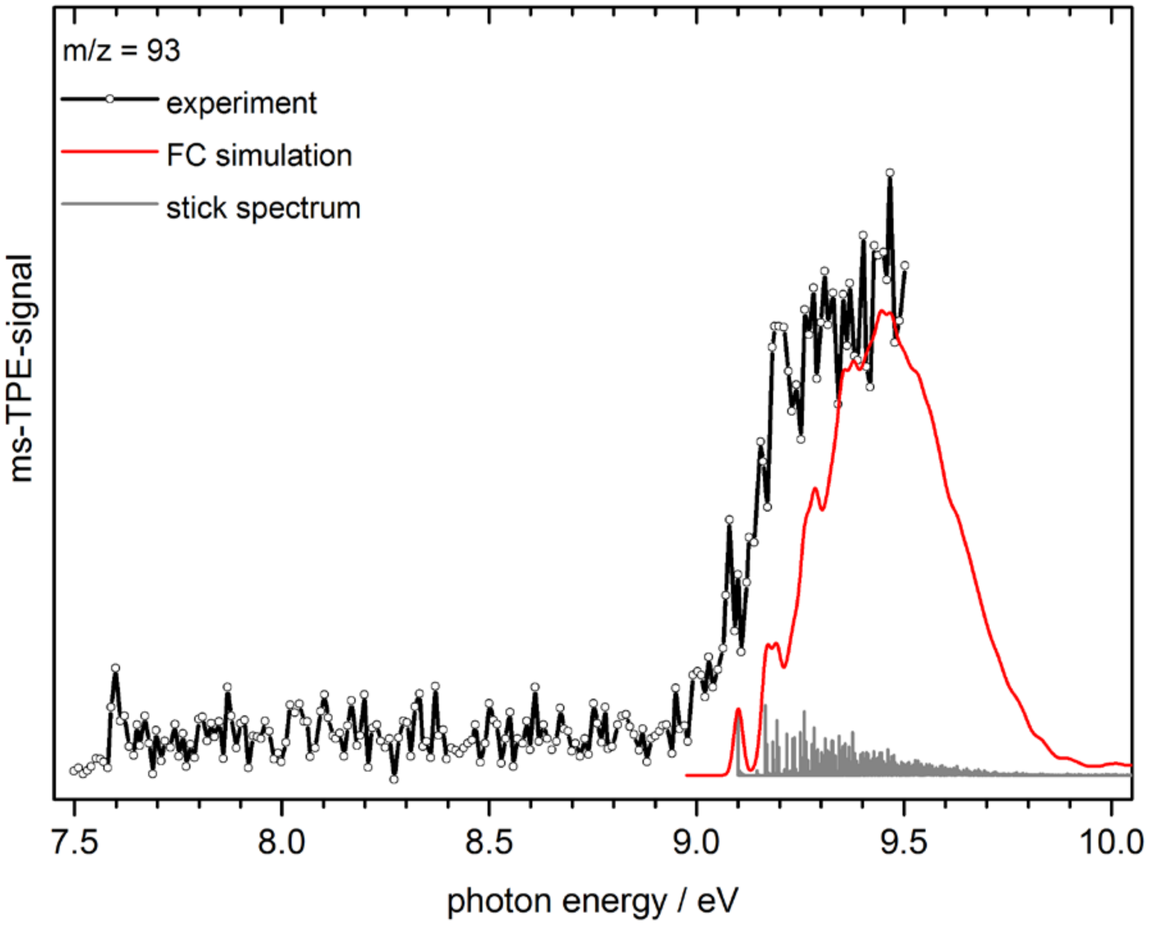

Figure S4. ms-TPE spectrum of *m*/*z* = 93 and a Franck-Condon simulation for 3-methylpyridine (red line), based on the CBS-QB3 level of theory.

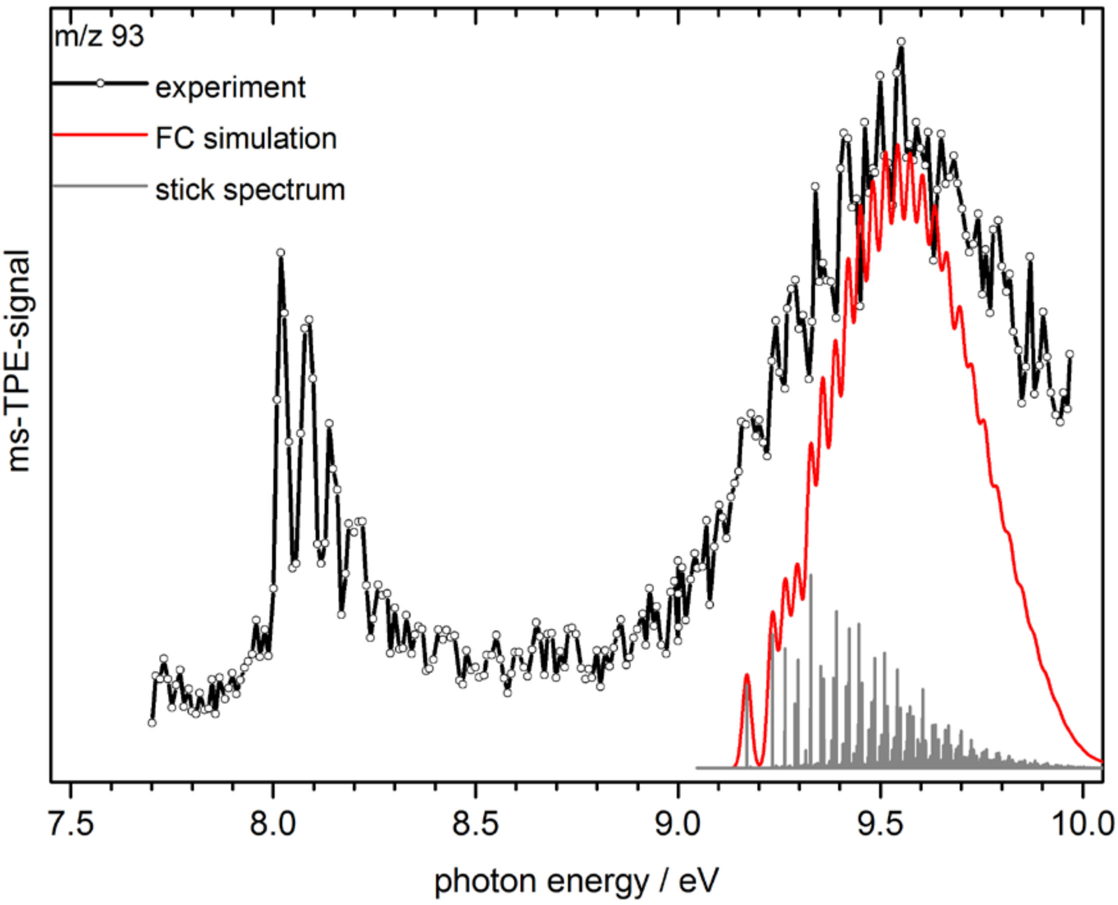

Figure S5. ms-TPE spectrum of *m*/*z* = 93 and a Franck-Condon simulation for 4-methylpyridine (red line), based on the CBS-QB3 level of theory. Around 8 eV signal from the ^{13}C isotopologue of picolyl appears.

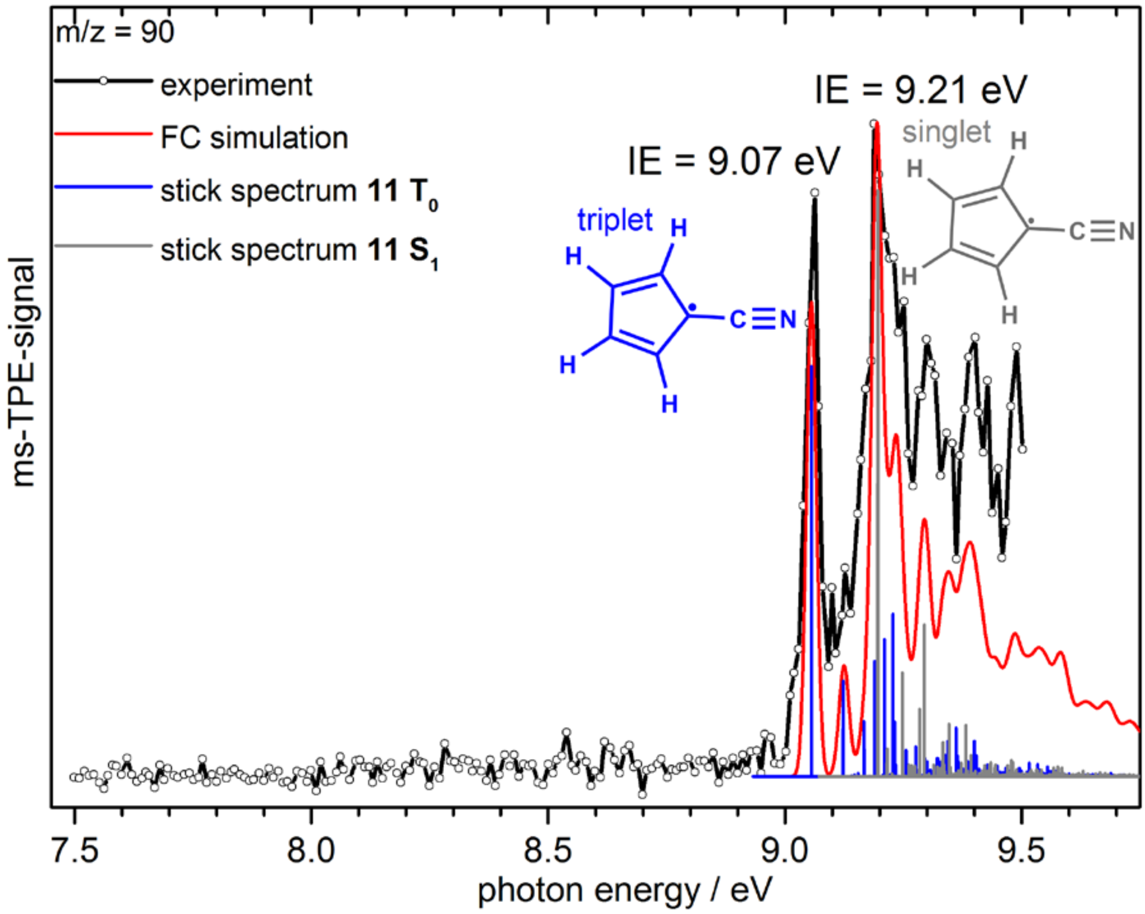

Figure S6. ms-TPE spectrum of *m/z* = 90, survey scan over an extended photon energy range from 7.50 to 9.50 eV. No signal from further isomers with *m/z* =90 is visible below 9.0 eV. The spectrum was obtained from precursor **2**.

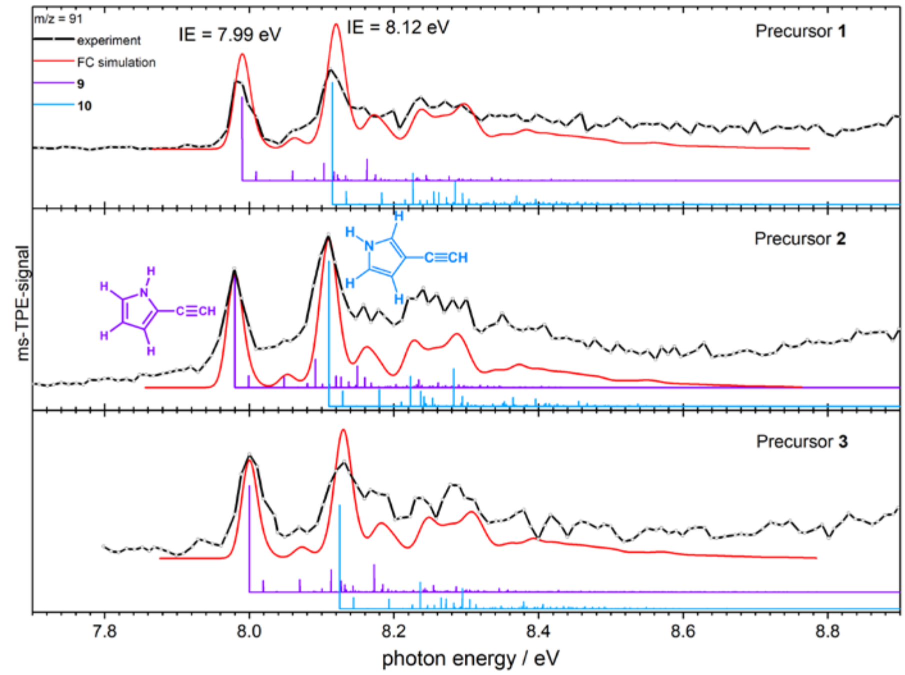

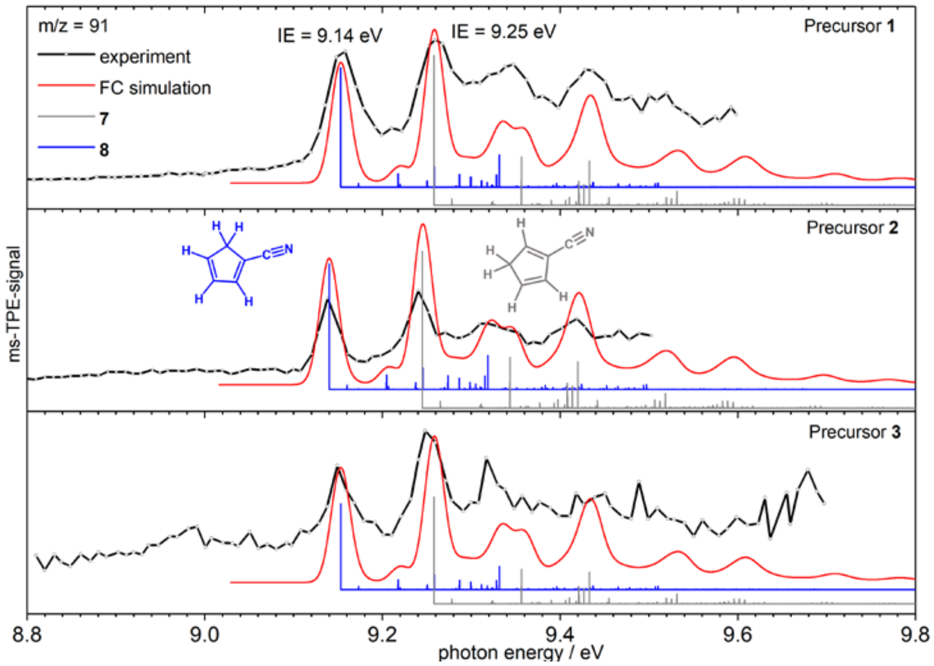

Figure S7. The mass-selected TPE spectra of m/z = 91 from all three precursors are very similar.

d) Absolute energies of 9 and 10 in comparison with aza-fulvenallenes.

Table S7: As visible aza-fulvenallenes are considerable less stable than ethynylpyrroles, which might explain their absence in the spectra. Energies were computed using CBS-QB3.

Name	Structure	Energy relative to **9** /kJ·mol^{-1}
**9**	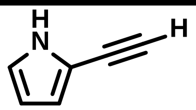	0
2-Vinylidene-2*H*-pyrrole	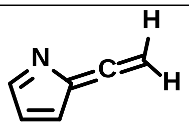	50.9
**10**	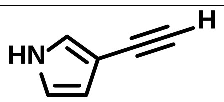	2.1
3-Vinylidene-3*H*-pyrrole	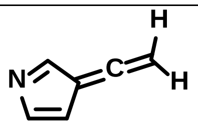	57.6

e) Illustration of the vibrational modes of 7, 8, 9 and 10.

Vibrational wavenumbers were computed using CBS-QB3.

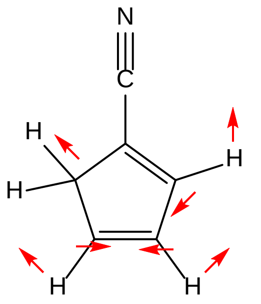

$\nu_8^+ = 1463\ \mathrm{cm}_{-1}$

cyclopenta-1,4-diene
-1-carbonitrile **7**

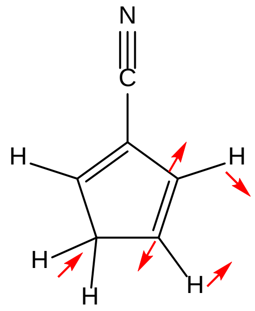

$\nu_8^+ = 1459\ \mathrm{cm}_{-1}$

cyclopenta-1,3-diene
-1-carbonitrile **8**

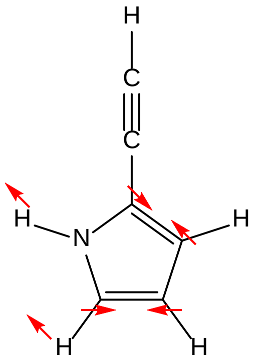

$\nu_9^+(a_1) = 1441\ \mathrm{cm}^{-1}$

2-ethynyl-1*H*-pyrrole **9**

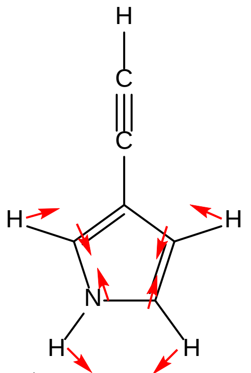

$\nu_9^+ = 1415\ \mathrm{cm}^{-1}$

3-ethynyl-1*H*-pyrrole **10**

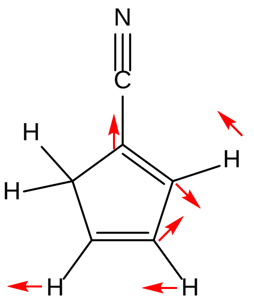

$\nu_9^+ = 1490\ \mathrm{cm}_{-1}$

cyclopenta-1,4-diene
-1-carbonitrile **7**

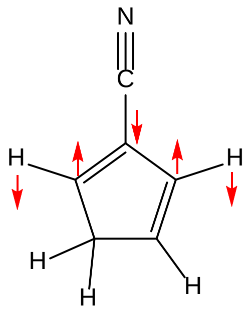

$\nu_9^+ = 1504\ \mathrm{cm}_{-1}$

cyclopenta-1,3-diene
-1-carbonitrile **8**

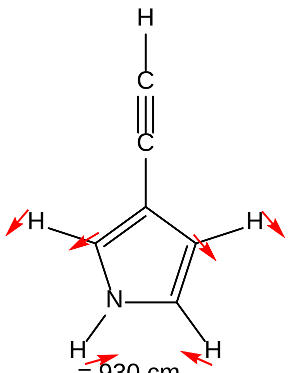

$\nu_{16}^+ = 930\ \mathrm{cm}_{-1}$

3-ethynyl-1*H*-pyrrole **10**

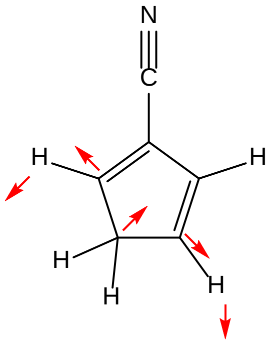

$\nu_{21}^+ = 822\ \mathrm{cm}_{-1}$

cyclopenta-1,3-diene
-1-carbonitrile **8**

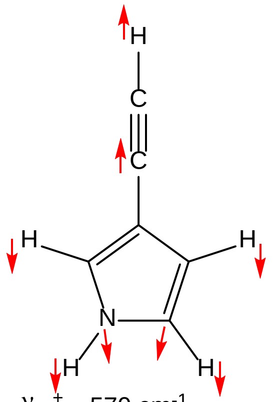

$\nu_{25}^+ = 570\ \mathrm{cm}^{-1}$

3-ethynyl-1*H*-pyrrole **10**

f) Table S8. Energies of stationary points along the reaction coordinate for the calculated decomposition pathway of 2-picolyl **4**, shown in scheme 2 of the main paper.

Name	Structure	Energy relative to **4** /kJ·mol^{-1}
**4**	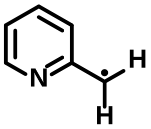	0
**IM1**	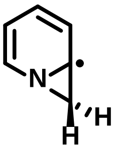	215.0
**IM2**	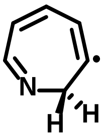	241.0
**IM3**	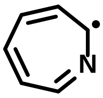	72.2
**IM4**	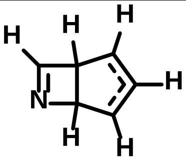	162.9
**IM5a**	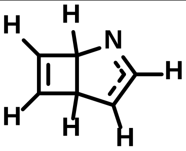	175.4
**IM5b**	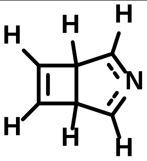	183.1
**IM6**	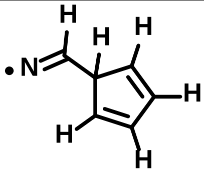	142.6
**IM7a**	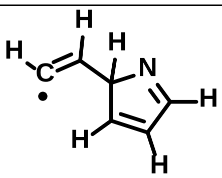	234.3
**IM7b**	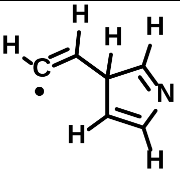	247.8

**IM8**	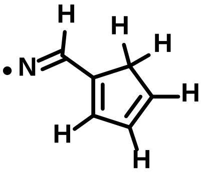	114.6
**IM9a**	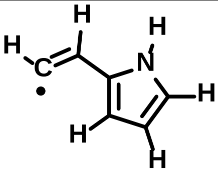	157.1
**IM9b**	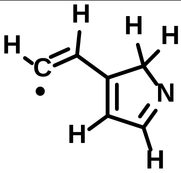	217.9
**IM10b**	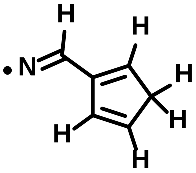	126.2
**IM11b**	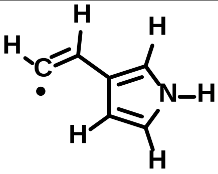	156.1
**7 + H**	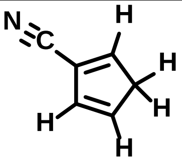 **+ H**	215.9
**8 + H**	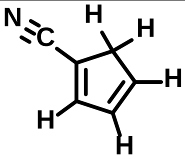 **+ H**	210.1
**9 + H**	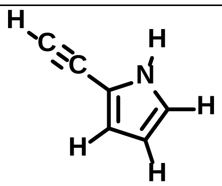 **+ H**	288.3
**10 + H**	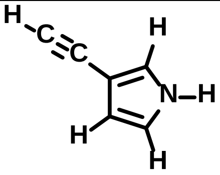 **+ H**	290.2
**12 + HCN**	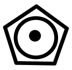 **+ HCN**	123.3

g) Further computed reaction pathways.

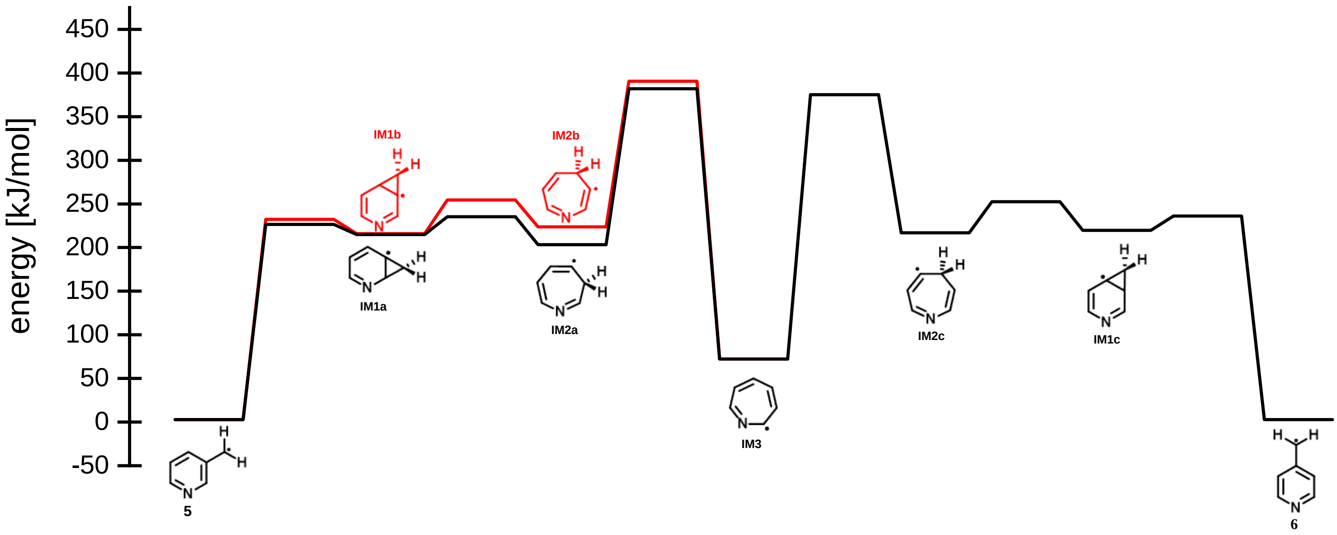

Figure S8. Reaction pathways of the 3-picolyl radical **5** (starting from the left) and 4-picolyl radical **6** (starting from the right) to the resonance stabilized azepinyl radical **IM3** computed by CBS-QB3. The 3-picolyl radical **5** has been set to zero, 4-picolyl was calculated to be 1 kJ/mol more stable.

Table S9. Energies of stationary points along the reaction coordinate, Figure S7.

Name	Structure	Energy relative to **9** /kJ·mol-1
**5**	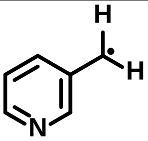	2.9
**6**	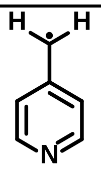	2.9
**IM1a**	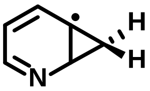	215.0
**IM1b**	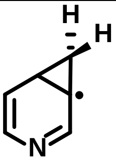	216.0
**IM1c**	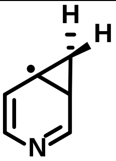	219.8
**IM2a**	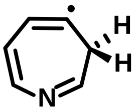	203.4
**IM2b**	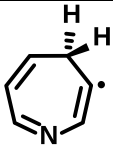	223.7
**IM2c**	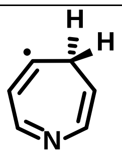	216.9
**IM3**	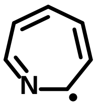	72.2

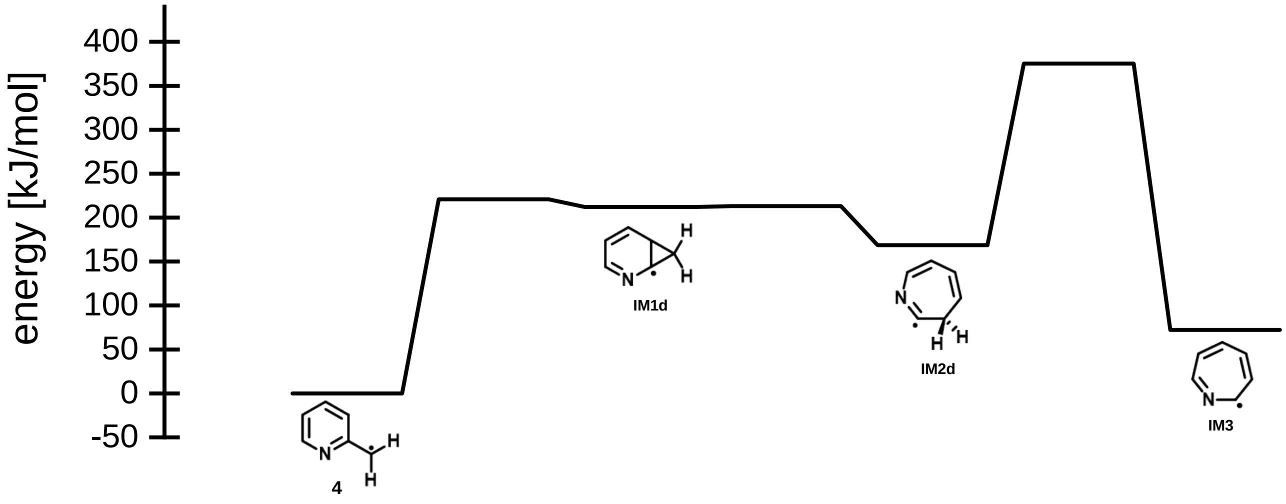

Figure S9. Alternative reaction pathway from **4** to the resonance stabilized radical **IM3**, computed by CBS-QB3. The energy of **4** has been set to zero.

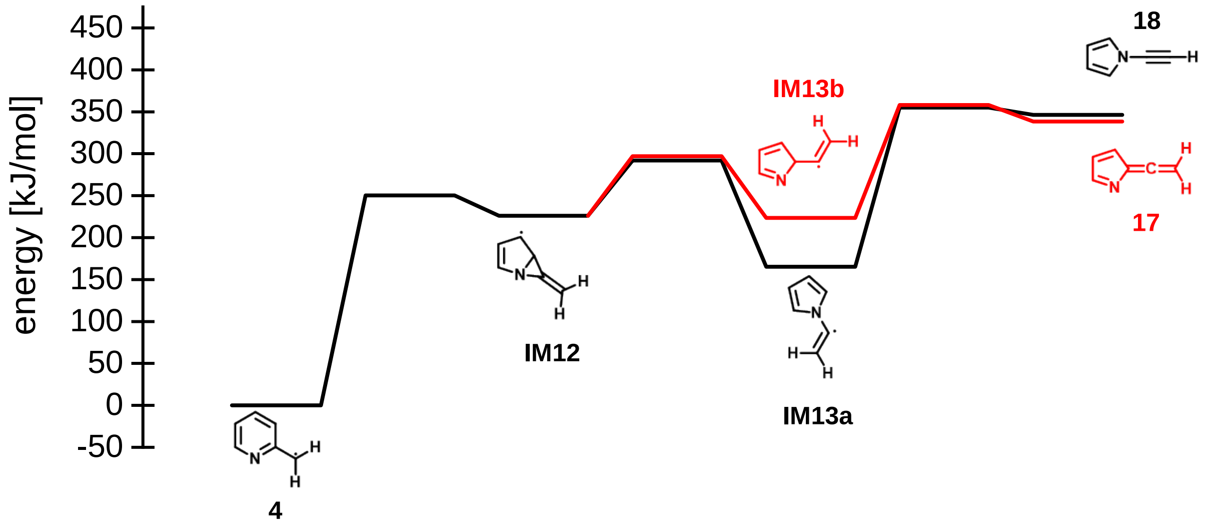

Figure S10 Reaction pathway from **4** to **17** and **18**, computed by CBS-QB3. The energy of **4** has been set to zero.
